# Controversies in metastatic hormone‐sensitive prostate cancer

**DOI:** 10.1002/cncr.70030

**Published:** 2025-08-07

**Authors:** Irene Tsung, Sarah E. Yentz, Zachery R. Reichert

**Affiliations:** ^1^ Division of Hematology/Oncology Department of Internal Medicine University of Michigan Ann Arbor Michigan USA

**Keywords:** bone modifying agent, controversies, metastatic hormone‐sensitive prostate cancer, PSMA PET, radiation, triplet therapy

## Abstract

Metastatic hormone‐sensitive prostate cancer (mHSPC) is an incurable phase of prostate cancer. Diagnostic tools and management strategies for this complex disease are expanding. Despite these advances, more therapies may not be the optimal approach for all patients. This review explores four major controversies surrounding mHSPC management—the role of prostate‐specific membrane antigen positron emission tomography scans as diagnostic imaging, triplet therapy (androgen deprivation therapy, androgen receptor pathway inhibitor, and docetaxel chemotherapy), radiation to the prostate and/or oligo‐metastases, and bone modifying agents. Critical evaluation of the data emphasizes the need for further work to determine which subgroups of patients with mHSPC benefit from each treatment. With a deeper understanding of these current issues, this review seeks to guide clinicians to refine their clinical practice to help patients achieve their best quantity and quality of life.

## Abstract 2

This review discusses key controversies in management of metastatic hormone‐sensitive prostate cancer, such as the roles of prostate‐specific membrane antigen positron emission tomography scans, triplet therapy, radiation, and bone‐modifying agents. It highlights the need to tailor treatments to specific patient subgroups for better outcomes.

## BACKGROUND

**FIGURE 1:**
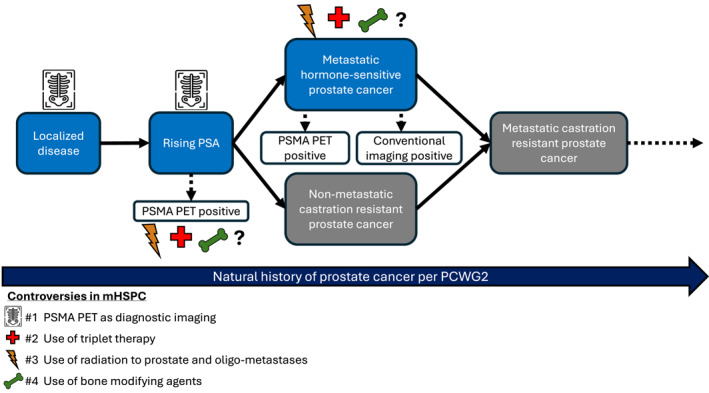
Natural history of prostate cancer based on PCWG2 and controversies in management of mHSPC. mHSPC indicates metastatic hormone‐sensitive prostate cancer; PCWG2, Prostate Cancer Working Group 2.

Prostate cancer is the second most frequently diagnosed cancer and fifth leading cause of cancer death among men globally in 2022, with an estimated 1.4 million new cases and nearly 400,000 deaths.^1^ By 2040, the incidence and deaths are projected to double.^2^ Although localized prostate cancer is often cured with radical prostatectomy or definitive radiation (with or without hormonal therapy), poor survival outcomes occur in patients who have recurrent (3%–8% of all comers) or de novo (∼10% at diagnosis) distant metastatic disease.^3^, ^4^, ^5^ The standard treatment for metastatic hormone‐sensitive prostate cancer (mHSPC) involves androgen deprivation therapy (ADT) and an androgen receptor pathway inhibitor (ARPI), with or without the addition of docetaxel chemotherapy and/or radiation.^6^ Treatment selection is complex due to the varied disease aggressiveness (for example, the control arm of ADT alone in three landmark trials had median overall survival [OS] of 54, 44, or 35 months depending on how patients chosen), prognosis, and older population often with competing noncancer health conditions.^4^ Understanding these complexities is essential because management decisions are not “one size fits all” and can influence patient outcomes, toxicities, and quality of life. Here, we explore controversies surrounding diagnosis, management, and treatment options in mHSPC (Figure 1).

## CONTROVERSY 1—DIAGNOSTIC IMAGING IN mHSPC

Mr. T is a 70‐year‐old man who presented with lower urinary tract symptoms and a prostate‐specific antigen (PSA) of 20 ng/mL. Prostate biopsy showed adenocarcinoma in all 14 cores with a maximum Gleason score of 4 + 3 (International Society of Urological Pathology [ISUP] Grade Group 3). A prostate‐specific membrane antigen (PSMA) positron emission tomography (PET) scan showed uptake in the prostate, multiple nonenlarged pelvic and retroperitoneal lymph nodes, and bones (left sixth rib, T2 and T10 vertebral bodies, and right ilium). The rib lesion did not have an anatomic correlate on computed tomography (CT).

Imaging to understand the extent of disease is crucial for determining therapy plans. Traditionally, conventional CT scans, magnetic resonance imaging (MRI), and technetium‐99m bone scans (BS) were used. However, since the 2020 approval by the US Food and Drug Administration (FDA) of the PSMA‐targeted radiotracers—Gallium 68 PSMA‐11 and piflufolastat F‐18—the routine use of PSMA PET has increased.^7^, ^8^ In a meta‐analysis of 31 studies with 2431 patients undergoing both PSMA PET and conventional imaging for initial staging of intermediate‐high risk prostate cancer, PSMA PET was more sensitive and specific for nodal and bone metastasis compared to CT (sensitivity 73.2% vs. 38.5%, specificity 97.8% vs. 83.6% for nodal; sensitivity 98.0% vs. 73.0%, specificity 96.2% vs. 79.1% for bone, respectively).^9^ For biochemically recurrent prostate cancer (BCR), the correct localization rate (CLR) was 85%–87% and the positive predictive value (PPV) was 92% with increasing rates of both at higher PSA levels. Even at low PSA <0.5 ng/mL, CLR was still 73.3%.^10^, ^11^ The CLR was defined as the PPV with the added condition of anatomic colocalization between the PSMA PET and a composite standard of truth (histopathology, subsequent correlative imaging findings, or post‐radiation PSA response). PSMA PET findings altered treatment plans for over half of patients.^10^, ^12^

**FIGURE 2:**
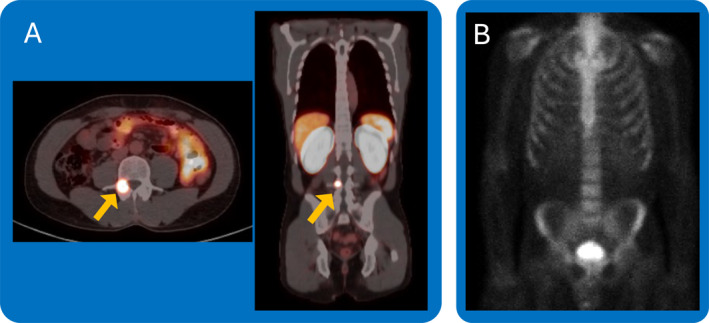
Discordant findings on PSMA PET versus technetium‐99m bone scan in a patient with de novo metastatic hormone sensitive prostate cancer. (A) Single bone lesion at the right L4 pedicle was PSMA‐avid on axial (left) and coronal (right) views. (B) Techneitum‐99m bone scan without uptake in the right L4 pedicle. How do you manage these discordant imaging findings? PET indicates positron emission tomography; PSMA, prostate‐specific membrane antigen.

The impressive diagnostic capabilities of PSMA PET scans have revolutionized prostate cancer staging and assessment of BCR. However, questions remain about their impact on outcomes:

- Does earlier detection and intervention with PSMA PET improve outcomes in an incurable disease state?
- With potential upstage migration using PSMA PET scans, should patients escalate treatment (Figure 2)? For Mr. T, does the rib lesion upstage his disease to CHAARTED high‐volume mHSPC (defined as presence of visceral metastases or four or more bone lesions with one or more outside the vertebral bodies and pelvis on conventional imaging)?^13^ With conventional imaging, this lesion and maybe others, could be normal.
- Do patients with PSMA PET only recurrences after definitive treatment have worse outcomes compared to patients with BCR by conventional imaging (that often detects metastases several years after PSMA PET identification)? Do patients with PSMA PET‐positive scans require immediate therapy?

PSMA PET scans improve disease detection, but the changes in clinical management on consequent oncologic outcomes are not established. For men with high pretest probability of metastatic disease by symptoms, PSA, and/or biopsy findings, starting with conventional imaging may be sufficient. We favor using PSMA PET for the initial staging in patients planned to undergo definitive prostatectomy because sparing surgical morbidity without likely surgical cure is prudent. PSMA PET for BCR may open treatment options such as oligometastatic‐directed radiation (discussed in controversy 3). Within historical BCR especially, the risk of earlier or more extensive systemic therapy should be considered. Indefinite hormonal therapy (as would be advised per SWOG 9346 lack of noninferiority)^14^ may be overtreatment as more therapy within BCR has not improved OS. The EMBARK study provides contemporary insights.^15^ Using traditional BCR radiographic criteria (CT/BS), higher risk patients (PSA doubling time ≤9 months and a PSA threshold ≥2 ng/mL after radiation or ≥1 ng/mL after prostatectomy) were randomized 1:1:1: to receive ADT + enzalutamide, ADT alone, or enzalutamide alone. To minimize toxicity, intermittent therapy was permitted for patients with a PSA response below 0.2 ng/mL at 9 months. The median PSA was 5.2 ng/mL with a doubling time of 5 months. A post hoc retrospective study of 182 patients with BCR by EMBARK criteria (not within the study) found that 46% had distant metastases on PSMA PET.^16^ Therefore, it is reasonable to extrapolate EMBARK outcomes to patients with molecular positive disease with similar PSA characteristics. EMBARK found improvements in 5‐year metastasis‐free survival with enzalutamide + ADT (87% vs. 71%; hazard ratio [HR], 0.42; 95% confidence interval [CI], 0.3–0.61) or enzalutamide alone (80% vs. 71%; HR, 0.63; 95% CI, 0.46–0.87) compared to ADT alone.^15^ Treatment breaks occurred in 91% with combination therapy, 68% with ADT, and 86% with enzalutamide, with grade ≥3 treatment‐related adverse events of 18%, 9%, and 16%, respectively. A substudy highlighted that enzalutamide monotherapy had the least impact on sexual function.^17^ Shared decision‐making should align treatment with individual preferences, such as treatment breaks, hot flashes, or sexual side effects. Currently, no high‐level evidence supports routine integration of PSMA PET to guide mHSPC treatment. Ongoing trials like ARASTEP (ClinicalTrials.gov identifier NCT05794906) and INDICATE (NCT04423211) are evaluating its impact on treatment planning for high‐risk BCR with PSMA PET‐positive but CT/BS‐negative lesions, like the EMBARK population. ARASTEP examines darolutamide + ADT versus placebo + ADT, whereas Arms C and D of INDICATE tests the addition of metastasis‐directed therapy (MDT) to apalutamide intensification and standard‐of‐care salvage radiation/ADT (in patients with PET positive extra‐pelvic metastases).

## CONTROVERSY 2—USE OF TRIPLET THERAPY FOR HIGH‐VOLUME mHSPC

Mr. M is a 79‐year‐old man with de novo mHSPC. His prostate biopsy showed 16 of 18 cores involved with adenocarcinoma with a maximum Gleason score of 5 + 4. Initial PSA was 62 ng/mL. Staging CTs showed multiple enlarged pelvic lymph nodes. Bone scan had uptake in the left iliac bone, sacrum, T11 vertebral body, and manubrium.

Adding an ARPI to ADT has transformed mHSPC treatment. Several randomized phase 3 trials—LATITUDE^18^ (abiraterone), STAMPEDE arm G^19^, ^20^ (abiraterone), TITAN^21^, ^22^ (apalutamide), and ARCHES^23^, ^24^ (enzalutamide)—show that ADT plus ARPI (without docetaxel) significantly reduces the risk of death by 34%–38% compared to ADT alone, regardless of disease burden.^21^, ^23^, ^25^ ENZAMET (enzalutamide + ADT vs. bicalutamide + ADT) was the only study with an active comparator and started to analyze the role for triplet therapy (ADT + ARPI + docetaxel) as 43% of patients had docetaxel.^26^, ^27^

Is three better then two? Rational combinatorial treatment strategies in oncology aim for additive or synergistic effects. The PEACE‐1^28^ and ARASENS^29^ trials evaluated triplet therapies, adding either abiraterone or darolutamide to ADT and six cycles of docetaxel in men with mainly de novo mHSPC (100% in PEACE‐1 and 86.1% in ARASENS). PEACE‐1 (*n* = 1173) was a multi‐arm trial that randomized patients to standard of care (SOC) systemic therapy, SOC plus radiation, SOC plus abiraterone, and SOC plus abiraterone and radiation. During the trial, SOC systemic therapy changed to allow both ADT monotherapy and ADT plus up‐front docetaxel with 60% of patients receiving docetaxel in addition to ADT (*n* = 710). When pooling groups for comparison (±radiation), the addition of abiraterone to SOC (ADT ± docetaxel) versus SOC without abiraterone improved median OS from 4.72 to 5.72 years (HR, 0.82; 95.1% CI, 0.69–0.98; *p* = .030). For only those who received docetaxel within SOC, abiraterone increased the median OS from 4.43 years to not reached (HR, 0.75; 95.1% CI, 0.59–0.95; *p* = .017). The median OS for high‐volume disease with triplet therapy (ADT, docetaxel, and abiraterone) was 5.14 years compared to 3.47 years with ADT + docetaxel (HR, 0.72; 95.1% CI, 0.55–0.95; *p* = .019). Low‐volume disease data were immature. The ARASENS trial (*n* = 1306) evaluated triplet therapy with darolutamide, ADT, and docetaxel compared to ADT and docetaxel. Darolutamide improved 4‐year OS to 62.7% from 50.4% (HR, 0.68; 95% CI, 0.57–0.80; *p* < .001) with post hoc subgroup analyses by metastatic burden (CHAARTED high 77% vs. low 23% in study) with the risk for death reduced in high‐volume (HR, 0.69; 95% CI, 0.57–0.82) but not low‐volume (HR, 0.68; 95% CI, 0.41–1.13).^30^ Similarly, the ENZAMET trial (*n* = 1125, 53% high‐volume, 47% low‐volume) showed survival benefit with the addition of enzalutamide to ADT plus docetaxel in de novo metastatic disease (HR, 0.73; 95% CI, 0.55–0.99), but its design did not focus on docetaxel’s effect, limiting interpretation. No benefit was seen in metachronous mHSPC. Regarding toxicity, PEACE‐1 and ARASENS both noted that adding ARPI to docetaxel/ADT did not increase incidence of severe or fatal adverse events, although ARASENS noted a rise in grade 2 peripheral sensory neuropathy with triplet therapy (9% from 3% SOC).

Several questions remain.

- How does triplet therapy compare to doublet therapy (ADT plus ARPI)? Because PEACE‐1 and ARASENS used ADT + docetaxel as comparators, the benefit of docetaxel is unclear.
- Should triplet therapy be applied to patients with recurrent/metachronous mHSPC, who generally have better prognosis? All patients in PEACE‐1 and most in ARASENs (86%) and ENZAMET (62%) enrolled de novo mHSPC, leaving its relevance to recurrent cases uncertain.

For abiraterone or darolutamide, the OS benefit was most pronounced in patients with de novo, high‐volume disease, and data was less impressive for those with low‐volume disease. In the case of Mr. M, it is reasonable to consider triplet therapy as he has high risk features and imaging consistent with high‐volume disease. However, factors such as his life‐expectancy, medical comorbidities (fitness, baseline neuropathy/risk of neuropathy), social support, and personal goals (e.g., chemo averse) should be considered in light of likely short term detriment and potential lifelong toxicities. We reserve triplet therapy for patients with de novo, high‐volume mHSPC who are clinically fit with low expectation for docetaxel intolerance, but better biomarkers may be on the horizon. *SPOP* mutations are associated with improved outcomes with ADT + ARPI,^31^ whereas triple‐negative tumor suppressor loss (*RB1*, *PTEN*, and *TP53*) are more dedifferentiated so may benefit more from upfront chemotherapy.^32^ PSA nadir ≤0.2 at 7 months is also a surrogate for improved OS and could lead to decisions regarding treatment intensification.^33^, ^34^

Randomized trials comparing triplet and doublet therapies are ongoing. ASPIRE is testing ADT and apalutamide with or without docetaxel (NCT06931340). A PSA‐directed study, TRIPLE‐SWITCH (NCT06592924), is evaluating delayed intensification of docetaxel in patients with suboptimal PSA response (PSA ≥0.2 ng/mL) after 6–12 months of ADT + ARPI. Both studies have tissue and molecular correlates that may become predictive.

## CONTROVERSY 3—USE OF RADIATION IN mHSPC

Mr. S is an 80‐year‐old man who presented with decreased urinary stream and PSA 20 ng/mL. Staging CT and BS showed three bone lesions (pelvis and spine).

Because Mr. S’ disease is outside the prostate and local lymph nodes, an important consideration is whether the prostate should be radiated.

In patients with low‐volume metastatic prostate cancer, prostate radiation might be considered. The STAMPEDE Arm H,^35^ HORRAD,^36^ and PEACE‐1^28^ trials evaluated its effect on OS. In STAMPEDE Arm H, patients with newly diagnosed metastatic prostate cancer were randomized to standard of care systemic therapy (primarily ADT monotherapy but 18% also received docetaxel) with or without radiation to the prostate. There was no survival benefit in the overall population (HR, 0.92; 95% CI, 0.80–1.06; *p* = .266), but in the prespecified subset of patients with low‐volume metastatic disease (40% of trial, *n* = 419), there was an OS benefit (HR, 0.68; 95% CI, 0.52–0.90; *p* = .007) and 3‐year survival benefit of 81% versus 73% in patients who received radiotherapy. Low volume disease was defined as someone who does not meet high volume criteria defined as per CHAARTED (see controversy 1). HORRAD (*n* = 432) was a similarly designed trial that also showed no benefit to radiation in the overall population but had a trend toward benefit in the subgroup of patients with less than five bone metastases (*n* = 160) (HR, 0.68; 95% CI, 0.42–1.10). PEACE‐1, described in controversy 2, also evaluated radiation, and in the low‐volume patients (*n* = 505), defined by the same criteria as in CHAARTED, OS was not improved. Median OS was 6.9 years (95.1% CI, 5.9–7.5) without radiation therapy (RT) versus 7.5 years (95.1% CI, 6.0–not reached) with RT (HR, 0.98; 95% CI, 0.74–1.28; *p* = .86).^37^ This trial discordance is likely due to improved systemic therapy in PEACE‐1 with 50% of patients receiving up‐front abiraterone (compared to 0% in other studies) and 50% receiving docetaxel (compared to 18% in STAMPEDE and 0% in HORRAD). Other end points were significant in PEACE‐1 such as a trend toward prolonged radiographic Progressoin Free Survival (HR, 0.65; 99% CI, 0.36–1.19; *p* = .02), time to emergence of castration resistance (HR, 0.62; 95% CI, 0.44–0.82), and time to serious genitourinary adverse events (log‐rank test; *p* = .003). Acute and late grade 3 or 4 bowel or bladder radiation toxicity was low at 5% or less.

Mr. A is a 66‐year‐old man with history of prostatectomy and salvage radiation 3 years ago for localized high‐risk prostate cancer with undetectable postoperative PSA. His PSA became detectable but has been slowly rising since then to 0.8 ng/mL. PSMA PET showed uptake in the left iliac crest and T12 vertebral body.

PSMA PET is increasingly detecting low‐volume, metachronous oligometastatic disease. This complicates treatment decisions because past trials were based on CT/BS staging. Along with PSMA PET, the use of MDT for recurrent mHSPC is rising. STOMP was a trial of 62 hormone‐sensitive patients with one to three metachronous metastases on choline PET imaging who were randomized to observation versus MDT of all sites. MDT improved 5‐year ADT‐free survival (34% vs. 8%; HR, 0.57; 95% CI, 0.38–0.84) but not OS (85% in both groups).^38^ ORIOLE was a similar trial evaluating a short‐term end point of progression at 6 months. In this trial, 54 men with one to three metachronous metastases on conventional imaging were randomized to MDT or observation. Progression at 6 months occurred in seven of 36 patients (19%) receiving stereotactic ablative body radiotherapy (SABR) and 11 of 18 patients (61%) undergoing observation (*p* = .005).^39^ Importantly, ORIOLE also found that those with untreated PSMA PET metastases (as they used conventional imaging to direct therapy) had significantly worse PFS than those who had all disease radiated (HR, 0.26; 95% CI, 0.09–0.75; *p* = .006), supporting the use of PSMA PET scans for therapy planning studies. The EXTEND^40^ trial differed from STOMP and ORIOLE by including intermittent hormone therapy. In this trial, 87 men with five or less disease sites were randomized to intermittent hormone therapy or intermittent hormone therapy plus MDT. Most were castrate sensitive (93%), received only ADT without ARPI (58%), and had disease defined by conventional imaging (75%). The median PFS for all participants was 22.4 months (95% CI, 17.7–not estimable) and was significantly longer in the combined therapy arm (not estimable) than in the hormone therapy only arm (15.8 months; 95% CI, 13.6–21.2).

A key question remains about managing systemic therapy alongside MDT. If MDT is pursued, can systemic therapy be eliminated or truncated?

PSMA PET has reclassified patients previously considered to have BCR as having metastatic disease. Historically, patients with BCR were managed with observation or intermittent therapy, as it was shown to be noninferior to continuous ADT with regard to OS and had fewer symptoms.^41^ Although intermittent therapy is still used, providers and patients may be more hesitant to take treatment breaks when metastases are visible on imaging citing SWOG 9346.^14^

The use of MDT (or intensified therapies as with EMBARK in controversy 1) might encourage more intermittent systemic therapy because treating all known disease sites with radiation can increase comfort with this approach. Regarding counseling on intermittent therapy, in SWOG 9346, disease assessment was based on conventional CT/BS and the threshold for restarting ADT was PSA level of 20 ng/mL. This is significantly higher than in contemporary trials such as EXTEND that used a PSA increase of only 2 ng/mL above nadir to resume ADT.

Overall, MDT’s added toxicity appears low. ORIOLE and STOMP reported no grade ≥3 adverse events. EXTEND showed similar grade 3 events between arms, although more grade 2 events occurred in the combination arm (12 vs. 4). Larger trials might reveal rarer toxicities, supporting ongoing studies.

Ongoing trials to clarify this area include: (1) STARPORT (NCT04787744), similar to EXTEND but using PSMA PET staging; (2) NRG‐GU011 (PROMETHEAN, NCT 05053152), which randomizes men with PET‐detected oligometastatic disease to SBRT plus 6 months of relugolix or SBRT plus placebo; and (3) SPARKLE (NCT05352178), a phase 3 trial randomizing oligo‐recurrent HSPC patients to MDT alone, MDT + 1 month ADT, or MDT + 6 months of ADT and enzalutamide.

In our practice, men with low‐volume, de novo mHSPC, prostate radiation should be considered, especially for those with local symptoms. We believe there is value in improved ADT‐free survival and PSA progression at 6 months. Our practice is to consider oligometastatic‐directed radiation, potentially with systemic therapy, depending on other disease characteristics. For example, for a patient like Mr. A with one to two recurrent disease sites, oligometastatic radiation may delay time to start of ADT. For someone with oligometastatic disease in five locations shortly after original definitive therapy, oligometastatic radiation plus intermittent systemic therapy seems preferred.

## CONTROVERSY 4—USE OF BONE MODIFYING AGENTS IN mHSPC

Mr. P is a 76‐year‐old man with de novo mHSPC. He presented with a PSA of 880 ng/mL. CT and BS revealed an enlarged prostate, numerous enlarged pelvic lymph nodes, and multiple bone lesions in the spine and ribs. There are no pathologic fractures noted. Dual‐energy X‐ray absorptiometry (DEXA) scan showed normal bone density with 10‐year probability of hip fracture 1% and major osteoporosis‐related fracture 7%. Should a bone modifying agent be initiated at this time?

Since recognizing that bone modifying agents (BMAs) reduce skeletal‐related events (SREs) in metastatic castration‐resistant prostate cancer (mCRPC), their benefits in mHSPC have been widely tested.^42^ It is important to focus on prostate cancer studies only as other cancers metastatic to bone act differently. For example, after a fracture, patients with prostate cancer, as opposed to breast cancer, did not have worse OS compared to those without a prior fracture (HR, 1.23; 95% CI, 0.96–1.57, *p* = .10).^43^ Overall, the use of BMAs in mHSPC is declining: 24% of 2627 treated mHSPC patients received a BMA between 2007 and 2015 versus 3% of 10,717 patients from 2017 to 2023 in separate studies.^44^, ^45^ Is this decline justified and should it become zero?

Some central tenets are necessary to place BMA studies into context. Original mCRPC studies showing morbidity benefit used BMAs without effective anticancer therapy, thus patients had growing bone metastases. That differs from mHSPC, where >90% of patients respond to therapy (>50% PSA reduction).^46^

BMAs in prostate cancer do not improve OS in mHSPC or mCRPC. Within mHSPC, STAMPEDE randomized patients to ADT alone or with zoledronic acid (ZA) monthly for 2 years started at a median of 8 weeks from ADT initiation without OS gains (HR, 0.9; 95% CI, 0.77–1.11) and had an osteonecrosis of the jaw (ONJ) rate of 2%–4%.^47^ CALGB 90202 randomized patients to monthly, indefinite ZA started within 6 months of ADT or placebo.^48^ At PSA progression (occurred in ∼50% of the control arm), control patients crossed over to ZA, thus testing early versus late use. It was negative for end points of time to first SRE (HR, 0.97) and OS (HR, 0.88; 95% CI, 0.7–1.12; *p* = .29). The rates of ONJ were 3.2% and grade ≥3 hypocalcemia 2%. The only positive mHSPC study for survival (secondary end point) accrued in the 1990s with 11 years of follow‐up and tested clodronate^49^ but it was negative for the primary end point of bone PFS.^50^ A meta‐analysis of the two ZA studies confirmed no OS improvement (HR, 0.94; 95% CI, 0.83–1.07; *p* = .323).^51^ Although studied in multiple contexts and even a randomized trial of 1468 mHSPC patients, a survival end point for denosumab (RANKL inhibitor) has not been reported.^52^

The use of BMAs in mHSPC may just reduce morbidity, as symptomatic fractures worsen mobility and increase risk for pain and other comorbidities.^53^ SRE prevention also failed for ZA in STAMPEDE for all comers (time to next skeletal event, HR, 0.89; 95% CI, 0.73–1.07; *p* = .221) and for those with bone metastases at baseline (HR, 0.94; 95% CI, 0.76–1.16).^47^ CALGB 90202 results were similar for all comers but suggested a benefit for those with prior SREs (*n* = 82, 13% of total cohort, HR, 0.56; 95% CI, 0.30–1.02; *p* = .54).^48^ The large denosumab mHSPC study (HALT) had secondary end points of SRE rates and randomized 1468 mHSPC patients to denosumab once every 6 months versus observation, a different dosing frequency than monthly in CRPC.^52^ At baseline, 22% of the intervention and 27% of the control arms had a history of SRE, and 76% of both arms had greater than 6 months of prior ADT exposure. The primary end point was percent change in bone mineral density at 24 months, which was improved. However, the clinical end point of fracture rate at any site was not statistically different between cohorts at 5.2% with denosumab and 7.2% with observation (HR, 0.72; 95% CI, 0.48–1.0; *p* = .10). It was statistically lower for vertebral fractures at 2.5% with denosumab and 3.9% with observation (HR, 0.38; 95% CI, 0.79–0.78; *p* = .006) but was likely driven by vertebral fractures seen on x‐rays (regardless of pain), so an unclear clinical relevance. Denosumab at this frequency is safer with similar total rates of AEs compared to placebo over 2 years, no ONJ cases seen, and a 0.1% hypocalcemia rate.

The all‐comer decline in BMA use aligns with the OS and SRE‐focused data but likely should not reach zero. For those without prior SRE events, screening for other osteoporosis risk factors and/or empiric DEXA testing to identify and treat osteoporosis (ZA annually or denosumab every 6 months) balances toxicity and benefit.^54^, ^55^ For those presenting with an SRE, maximizing bone health is reasonable because they are at higher risk of future SREs. Treating with prior mCRPC frequencies like STAMPEDE or CALGB 90202 may not be needed. We often do four doses at 12‐week intervals of ZA (shown to be equivalent to monthly dosing in mCRPC with only a 1% rate of ONJ over 2 years) and then 1 year of annual osteoporosis dosing.^56^ After those two years, they are managed similarly to those without a prior SRE with DEXA scan screenings every 2 years. Less data for alternative denosumab frequencies exists, so for those who are not candidates for ZA, osteoporosis dosing intervals seem fine. For Mr. P, our practice is to hold empiric BMA and screen by DEXA first.

## CONCLUSION

The landscape for management of mHSPC continues to evolve with new technologies and treatments. The data for using these must be critically evaluated before integration into daily practice. Caution must be taken to not overtreat patients without clear evidence of benefit. After all, more is not always better.

## AUTHOR CONTRIBUTIONS

**Irene Tsung:** Conceptualization, data curation, formal analysis, methodology, resources, writing–original draft, and writing–review and editing. **Sarah E. Yentz:** Conceptualization, data curation, investigation, methodology, resources, writing–original draft, and writing–review and editing. **Zachery R. Reichert:** Conceptualization, data curation, formal analysis, investigation, methodology, resources, writing–original draft, and writing–review and editing.

## CONFLICT OF INTEREST STATEMENT

Zachery Reichert reports advisory roles for AstraZeneca and Janssen; and research funding through the institution from AstraZeneca. The other authors declare no conflicts of interest.
